# A flow-cytometry-based pipeline for the rapid quantification of C2C12 cell differentiation

**DOI:** 10.1016/j.xpro.2023.102637

**Published:** 2023-10-10

**Authors:** Bianca Parisi, Maxime Sünnen, Rohan Chippalkatti, Daniel Kwaku Abankwa

**Affiliations:** 1 Cancer Cell Biology and Drug Discovery Group, Department of Life Sciences and Medicine, University of Luxembourg, 4362 Esch-sur-Alzette, Luxembourg

**Keywords:** Cell Biology, Cancer, Molecular Biology

## Abstract

The C2C12 cell line represents a simple *in vitro* model for cell differentiation. Here, we present a flow-cytometry-based pipeline to quantitate C2C12 cell differentiation based on myosin heavy-chain marker expression. We describe steps for cell seeding, transfection, drug treatment, differentiation, and labeling. We then detail procedures for flow cytometry acquisition and introduce the R script FlowFate for automated analysis, including the study of dose-dependent effects of GFP-tagged genes on differentiation.

For complete details on the use and execution of this protocol, please refer to Chippalkatti et al. (2023).^1^

## Highlights

• Preparation of transfected or drug-treated C2C12 cells for flow cytometry
• C2C12 cell differentiation data acquisition on a flow cytometer
• R-based FlowFate for automated analysis of GFP-construct differentiation data

## Abstract (editor highlights)

Publisher’s note: Undertaking any experimental protocol requires adherence to local institutional guidelines for laboratory safety and ethics.

## Graphical abstract

**Figure:**
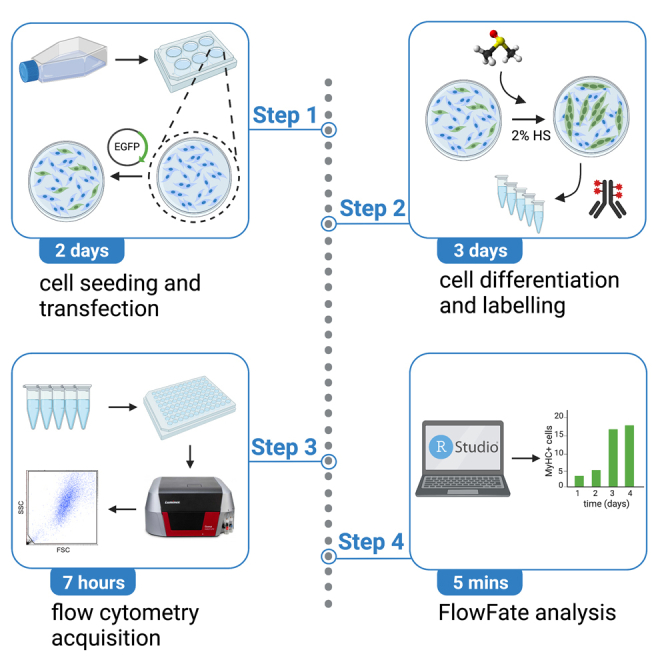

## Before you begin

Cell differentiation is a fundamental process during development, but also highly relevant in regenerative research and for understanding the evolution of cancer and other diseases. Only a few *in vitro* models exist that faithfully recapitulate essential facets of cell differentiation as observed *in vivo*.^2^

The heterogeneous mouse muscle C2C12 myoblasts proliferate under high serum (10% fetal bovine serum) conditions.^3^ Upon switching to low serum (2% horse serum), a fraction of these cells differentiate and after typically 3 days the late differentiation marker myosin heavy chain (MyHC) is expressed.^4^ Using flow cytometry, we here quantify the fraction of cells that expresses MyHC as a measure of C2C12 cell differentiation.

**Figure 1:**
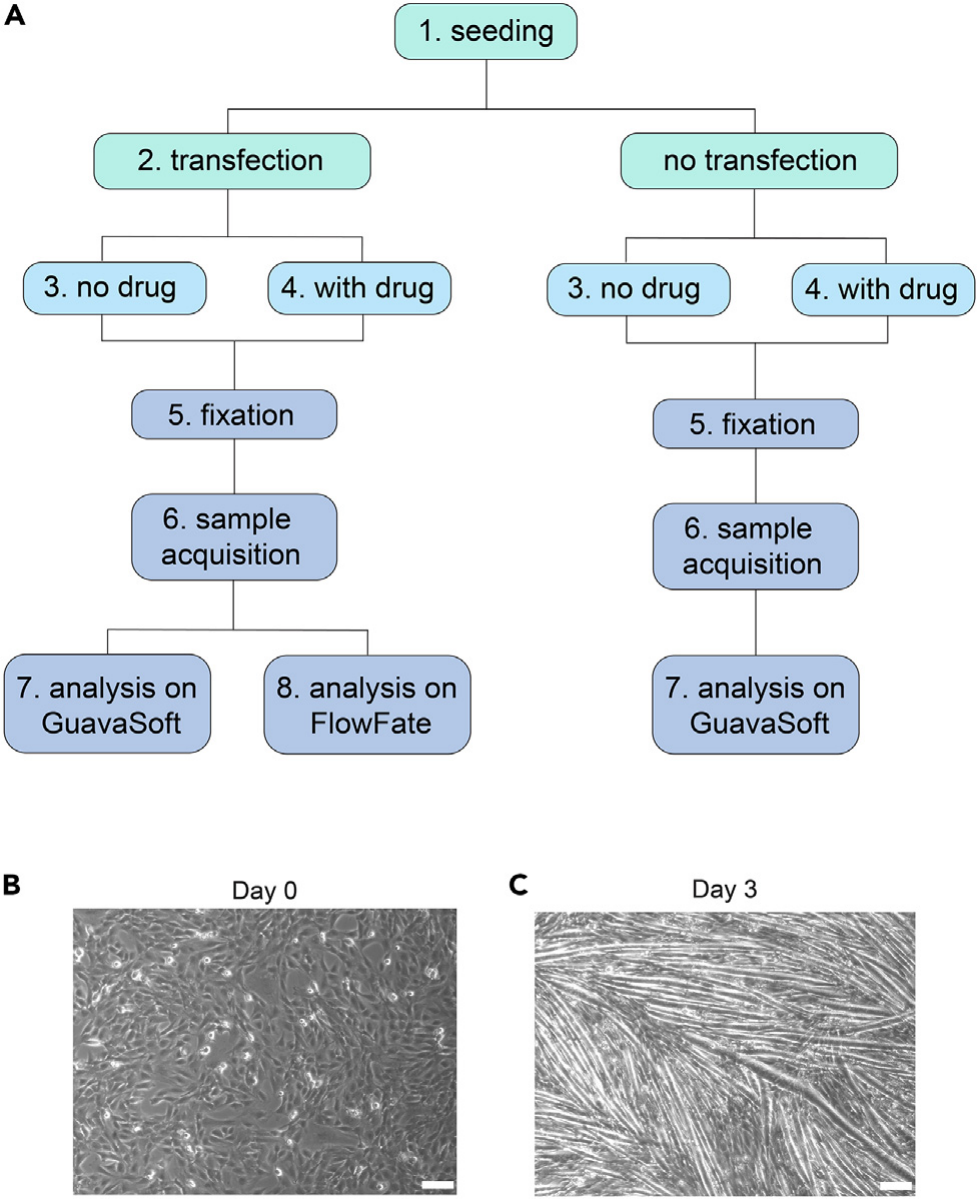
Overview of differentiation phenotype and analysis pipeline (A) Flowchart of experimental and analysis steps with part-numbering as applied in this protocol. (B) C2C12 cells at ∼90% confluency in high serum medium. (C) Differentiated myotubes after culturing cells in low serum medium for 3 days. Scale bar 200 μm (B, C).

We describe how this assay can quantify the extent to which GFP-variant tagged disease mutants, in our case of the oncogene *KRAS*, impact differentiation. Gating for GFP-positive cells allows to analyze specifically the mutant expressing subpopulation in a gene-dose dependent manner. We furthermore demonstrate how drug treatments can rescue this aberrant activity. Both GFP-construct expression and drug treatments are optional and the flowchart of our step-by-step experimental and analysis pipeline illustrates these options (Figure 1A).

Our assay is the most quantitative assay of C2C12 cell differentiation to date and uniquely allows to measure the impact of transient genetic manipulations in combination with drug treatments at a single-cell level. It may thus emerge as an important new tool in cancer research that enables the analysis of the differentiation restoring activity of drugs, as compared to the common anti-proliferative or cell viability assays.

1. To get started, prepare the necessary media based on the recipes cataloged in the materials and equipment section.
2. All solutions and buffers required in this protocol can be either freshly prepared on the day of the experiment or in advance and subsequently stored as described.

## Key resources table

**Table:** 

REAGENT or RESOURCE	SOURCE	IDENTIFIER
**Antibodies**		

Myosin 4 monoclonal antibody (MF20), eFluor 660, eBioscience (dilution 1:100)	Thermo Fisher Scientific	Cat# 50-6503-82
PE anti-HA tag antibody (16B12) (dilution 1:20)	Abcam	Cat# ab72479

**Chemicals, peptides, and recombinant proteins**		

Dulbecco’s phosphate-buffered saline (PBS) (1×)	Thermo Fisher Scientific	Cat# 14040091
Dulbecco’s modified Eagle’s medium (DMEM)	Thermo Fisher Scientific	Cat# 41965039
Trypsin EDTA (0.05%)	Thermo Fisher Scientific	Cat# 25300054
Fetal bovine serum (FBS)	Thermo Fisher Scientific	Cat# 10270106
Horse serum (HS)	Thermo Fisher Scientific	Cat# 16050130
Penicillin-Streptomycin (10,000 U/mL)	Thermo Fisher Scientific	Cat# 15140122
L-glutamine (200 mM)	Thermo Fisher Scientific	Cat# 25030024
Dimethyl sulfoxide (DMSO) ≥99.5%	VWR	Cat# A3672
Paraformaldehyde (PFA) 16% (w/v)	Thermo Fisher Scientific	Cat# 43368.9M
Tween 20	Merck	Cat# P2287
Triton X-100	Merck	Cat# T8787
ISOTON II diluent	Beckman Coulter	Cat# 8448011
Guava easyCheck Beads	Cytek Biosciences	Cat# 4500-0025
AMG 510/sotorasib	MedChemExpress	Cat# HY-114277

**Critical commercial assays**		

jetPRIME	Polyplus-transfection	Cat# 101000001

**Experimental models: Cell lines**		

C2C12 mouse cell line	American Type Culture Collection	Cat# CRL-1772

**Software and algorithms**		

GuavaSoft 4.0 Software	Cytek Biosciences	N/A
Microsoft Excel	Microsoft Corporation	N/A
GraphPad Prism version 9.5.1	GraphPad Software	N/A
Adobe Illustrator version 26.0.1	Adobe Systems, Inc.	N/A
BioRender	BioRender.com	N/A
R version 4.3.0	R foundation for statistical computing^5^	https://cran.rstudio.com/
RStudio version 2022.12.0+353	RStudio^6^	https://posit.co/download/rstudio-desktop/
flowCore version 2.12.0	Hahne et al.^7^	https://bioconductor.org/packages/release/bioc/html/flowCore.html
flowWorkspace version 4.12.0	Finak et al.^8^	https://www.bioconductor.org/packages/release/bioc/html/flowWorkspace.html
openCyto version 2.12.0	Finak et al.^9^	https://bioconductor.org/packages/release/bioc/html/openCyto.html
ggcyto version 1.28.0	Van et al.^10^	https://www.bioconductor.org/packages/release/bioc/html/ggcyto.html
FlowFate version 1.2	This study	https://doi.org/10.5281/zenodo.8325331

**Other**		

CO_2_ incubator	Panasonic	Cat# MCO-170AICUVL-PA
Guava easyCyte 6HT 2L flow cytometer	Cytek Biosciences	N/A
Microcentrifuge Micro Star 17R	VWR	Cat# 521-1647
Z1 particle counter	Beckman Coulter	Cat# 9914591
Cuvette Coulter	VWR	Cat# 720-0812
T75 flask	Greiner Bio-One	Cat# 658175
Eppendorf 1.5 mL	Greiner Bio-One	Cat# 616201
Falcon 15 mL	Greiner Bio-One	Cat# 188271
Falcon 50 mL	Greiner Bio-One	Cat# 227261
6-well plate	Greiner Bio-One	Cat# 657160
96-well plate	Greiner Bio-One	Cat# 655180

## Materials and equipment

• Flow cytometer configuration.

Here, a Guava easyCyte 6HT 2L flow cytometer was used to acquire flow cytometry data. The instrument is equipped with 50 mW photodiode 488 nm and 100 mW photodiode 642 nm laser lines. The following filters were employed in the instrument configuration: forward scatter (FSC) and side scatter (SSC) (band pass 488/16), Green-B (band pass 525/30) and Red-R (band pass 662/15). The emitted light was detected using photodiode detectors in case of FSC and SSC and photomultiplier tubes for all other channels. mEGFP was detected with 488 nm laser excitation and the Green-B filter. MyHC was detected after labeling with a Myosin 4 monoclonal antibody (MF20) conjugated to eFluor 660 (excitation maximum 633 nm and emission maximum 669 nm) using the 642 nm laser and the Red-R filter.

• High serum culture medium.

**Table:** 

Reagent	Final concentration	Amount
DMEM	1×	500 mL
Fetal Bovine Serum (FBS)	∼9% (v/v)	50 mL
Penicillin-Streptomycin solution	1% (v/v)	5 mL
L-glutamine	2 mM	5 mL
**Total**	**N/A**	**560 mL**

Store at 4°C for up to 1 month.

• Low serum culture medium.

**Table:** 

Reagent	Final concentration	Amount
DMEM	1×	500 mL
Horse Serum (HS)	∼2% (v/v)	10 mL
Penicillin-Streptomycin solution (10,000 U/ mL)	1% (v/v)	5 mL
L-glutamine	2 mM	5 mL
**Total**	**N/A**	**520 mL**

Store at 4°C for up to 1 month.

• PBS-T.

**Table:** 

Reagent	Final concentration	Amount
Phosphate buffered saline (PBS)	N/A	50 mL
Tween 20	0.05% (v/v)	25 μL
**Total**	**N/A**	**50.025 mL**

Store at 4°C for up to 1 month.

• 4% paraformaldehyde (PFA) fixation solution.

**Table:** 

Reagent	Final concentration	Amount
PBS	N/A	30 mL
Paraformaldehyde (PFA) 16% (w/v)	4% (w/v)	10 mL
**Total**	**N/A**	**40 mL**

Store at 4°C for up to 1 month.

• 0.5% Triton X-100 in PBS.

**Table:** 

Reagent	Final concentration	Amount
PBS	N/A	50 mL
Triton X-100	0.5% (v/v)	250 μL
**Total**	**N/A**	**50.25 mL**

Store at 22°C–25°C for up to 1 month.

## Step-by-step method details

### Part 1: Cell seeding

**Timing: 60 min**

This section describes the typical procedure for passaging and maintaining C2C12 cells.

1. Prewarm high serum medium, PBS and trypsin EDTA at 37°C in a water bath for at least 5–10 min before the beginning of the experiment.
2. Turn on the Beckman Coulter Z1 Counter for cell counting and flush it twice with MilliQ water and twice with ISOTON II Diluent before use.
3. Culture C2C12 cells in a T75 flask under standard cell culture conditions in high serum culture medium (37°C, 5% CO_2_) until they reach a confluency of 50%–60%.
4. Remove the high serum medium from the T75 flask.
5. Gently wash the cells once with 10 mL sterile PBS.
6. Aspirate the PBS and add 5 mL 0.05% trypsin EDTA and incubate at 37°C until the cells have detached (5–7 min).
7. Resuspend cells in 5 mL high serum medium to neutralize the trypsin and pipette carefully up-and-down to separate cells.
8. Transfer the suspension to a 15 mL falcon tube and centrifuge at 200 × *g* for 3 min to pellet the cells.
9. Discard the supernatant and resuspend the pellet in 1 mL fresh high serum medium into a single-cell suspension by carefully pipetting up-and-down.
10. Dilute 50 μL of the cell suspension into 10 mL of ISOTON II Diluent and measure the cell concentration on the Z1 particle counter.
11. Adjust to the final cell concentration of 50,000 cells/mL.

***Note:*** Use the following calculation C_1_×V_1_ = C_2_×V_2_. C_1_ and V_1_ are the initial concentration and volume, respectively, and C_2_ and V_2_ are the final concentration and volume, respectively.

12. Resuspend the accurate number of cells in high serum medium and plate 2 mL of cell suspension in each well of 6-well plates to obtain the final density of 100,000 cells per well.
13. Move the plate back and forth and sideways and allow cells to settle evenly in 6-well plates for around 20 min outside the incubator to avoid cell aggregation at the center of the well.
14. Incubate cell culture plates at standard cell culture conditions until the desired cell confluency is obtained (50%–60% for transfection and 90% for serum switching to low serum medium).
15. Continue to part 2 to analyze the effect of a GFP-variant tagged gene of interest. For analysis of non-transfected cells continue to part 3 (no drug treatment) or part 4 (with drug treatment).

### Part 2. Cell transfection with a GFP-variant tagged gene of interest

**Timing: 30 min**

This part is optional and can be applied to investigate the impact of disease variant genes of interest and the gene dose on C2C12 cell differentiation. It can be combined with drug treatments to furthermore assess a drug’s effect on the transiently genetically altered C2C12 cells (part 4). C2C12 cell transfection with a GFP-variant tagged gene of interest can be performed once the cells have reached 50%–60% confluency. Alternatively, a construct coexpressing a GFP-variant using an IRES-element or P2A-autocleavage sequence may be employed, however, this approach may require additional validations (see Expected outcomes section).

The following volumes are optimized for the transfection of cells cultured in a 6-well plate, using jetPRIME transfection reagent according to the manufacturer’s instructions (http://tinyurl.com/5ejcwmst). Other transfection reagents such as Lipofectamine 2000 can be used instead of jetPRIME. All handling should be carried out under sterile conditions inside a laminar flow hood.

16. Dilute 2 μg plasmid DNA into 200 μL jetPRIME buffer and mix by vortexing.
17. Vortex jetPRIME reagent for 5 s and spin down shortly before use.
18. Add 4 μL of jetPRIME reagent to the transfection solution containing DNA, mix by vortexing and spin down shortly.
19. Incubate the transfection mix for 10 min at 22°C–25°C.
20. Add 200 μL of transfection mix dropwise per well with 2 mL medium and distribute evenly.
21. Replace medium 4 h after transfection with fresh high serum medium.
22. For the analysis of cells transfected with a mEGFP-tagged gene of interest continue to part 3 (no drug treatment) or part 4 (with drug treatment).

**CRITICAL:** Incubating cells with transfection mix for longer times may result in toxicity. Other GFP-variants than mEGFP may be employed, such as EGFP or even EYFP. However, in that case, all control and target samples need to carry this GFP-variant.

### Part 3. Cell differentiation without drug treatment

**Timing: ∼10 min each day for 3 days**

In part 3 we describe the C2C12 cell differentiation after switching to low serum culture conditions. Switching to low serum medium should be carried out once the cells have reached 90% confluency (Figure 1B). For drug treatments during cell differentiation, please refer to the next part 4.

23. One day after transfection (optional) replace high serum medium with prewarmed low serum medium.
24. Add fresh low serum medium every day for 3 days to allow cells to differentiate into myotubes (Figure 1C). Continue to part 5.

### Part 4. Cell differentiation with drug treatment

**Timing: ∼30 min each day for 3 days**

This part describes the essential steps to evaluate the effect of drug treatment on differentiation.

Equilibrate the stock of the drug, here AMG 510/ sotorasib, at 22°C–25°C. Any other drug or experimental compound can be tested in the same way.

25. Prepare working solutions of the desired final drug concentrations in prewarmed low serum medium.
26. One day after transfection (optional), replace high serum medium with 2 mL of working solution to each well.
27. Add fresh working solution every day for 3 days. Continue to part 5.

**CRITICAL:** The final DMSO concentration must be ≤0.2% to avoid effects on differentiation. For other solvents, the concentration that does not impact on differentiation has to be determined essentially using the procedure described here in part 4.

### Part 5. Cell fixation, permeabilization and MyHC-immunolabeling

**Timing: 2–3 h**

This section provides details on performing antibody labeling for the samples.

28. Remove the low serum medium, add 750 μL 0.05% trypsin EDTA and incubate at 37°C for 2 min and immediately start pipetting and detaching the cells.
29. Resuspend cells in 750 μL low serum medium and pipette carefully up-and-down to separate cells.
30. Transfer each cell suspension into a 1.5 mL Eppendorf tube and centrifuge at 500 × *g* for 5 min to pellet the cells.
31. Discard the supernatant and wash the cells once with 500 μL PBS.
32. After another round of centrifugation at 500 × *g* for 5 min, discard the supernatant and fix with 500 μL 4% PFA. Incubate for 10 min on a shaker at 22°C–25°C.
33. Centrifuge the samples at 500 × *g* for 5 min, discard the supernatant and resuspend the pellet with 500 μL PBS. Samples can be stored at this stage for up to 1 week at 4°C.
34. Centrifuge the samples at 500 × *g* for 5 min, discard the supernatant and permeabilize the cells with 500 μL 0.5% Triton X-100 in PBS for 10 min on a shaker.
35. Wash the samples with 500 μL PBS-T to remove excess of Triton X-100 and centrifuge them at 500 × *g* for 5 min.
36. Immunolabel the cells with 50–100 μL of Myosin 4 Monoclonal Antibody (MF20) conjugated to eFluor 660, which detects MyHC protein, at a dilution of 1:100 in PBS-T for 30 min at 4**°**C.

**CRITICAL:** Cover the samples with aluminum foil to prevent any light-induced bleaching during the incubation period.

37. Pellet the cells by centrifugation at 500 × *g* for 5 min and discard the supernatant.
38. Resuspend the cells in 500 μL of PBS for flow cytometry analysis. Acquire all samples on Guava easyCyte 6HT 2L flow cytometer.

### Part 6. Channel settings and sample acquisition on a flow cytometer

**Timing: ∼2 h**

Steps for calibration of the flow cytometer and setting up of channels are described here.

Include in each experiment controls for adjusting instrument settings that are required for the manual (part 7) or our automated gating and differentiation data analysis procedures (part 8).

39. The ‘double-negative control’ (MyHC-/GFP-) sample where C2C12 cells were not transfected, cultured under high serum conditions and immunolabeled with Myosin 4 monoclonal antibody (MF20) conjugated to eFluor 660.
40. The ‘GFP+ control’ is a C2C12 cell sample that expresses only a construct with mEGFP that is not genetically fused to another protein; required if GFP-variant constructs are investigated (part 2).
41. The ‘MyHC+ control’ is a 3-day differentiated C2C12 cell sample without GFP-construct expression labeled with Myosin 4 monoclonal antibody (MF20) conjugated to eFluor 660.

***Note:*** As there is no spectral overlap between GFP and eFluor 660, no compensation was performed in our examples. The Guava 6HT-2L flow cytometer can acquire samples contained either in 1.5 mL Eppendorf tubes or 96-well plates. To simplify acquisition of multiple samples and to conserve sample volume, we describe in this protocol only the acquisition of samples using the 96-well sampler of the instrument.

42. Then in addition to these controls, prepare any number of target samples that contain GFP+ and MyHC+ subpopulations for differentiation analysis.
43. Run the Guava Clean and then easyCheck quality control procedure by preparing a 1:20 dilution of Guava easyCheck Beads.

***Note:*** The Guava Clean procedure is instrument specific and done as described by the manufacturer to clean and unclog the instrument microfluidics before use. The easyCheck procedure calibrates the system and tests sensitivity of the optical components (lasers and detectors). Optimum system performance is validated if the fluorescence intensities in all channels and the beads count generated during this procedure fall within the range specified by the manufacturer. When using other flow cytometers prepare the instrument correspondingly.

44. Once the device is ready to use, set the number of events to be acquired to 30,000 and if needed the time of acquisition per well to 150 s. The acquisition time can be adjusted depending on the cell concentration.
45. Dispense 200–250 μL of each control sample and target sample into different wells of a 96-well plate.

***Note:*** Check sample concentration and dilute to obtain less than 500 events/ μL in order to avoid clogging of the microcapillaries.

**Figure 2:**
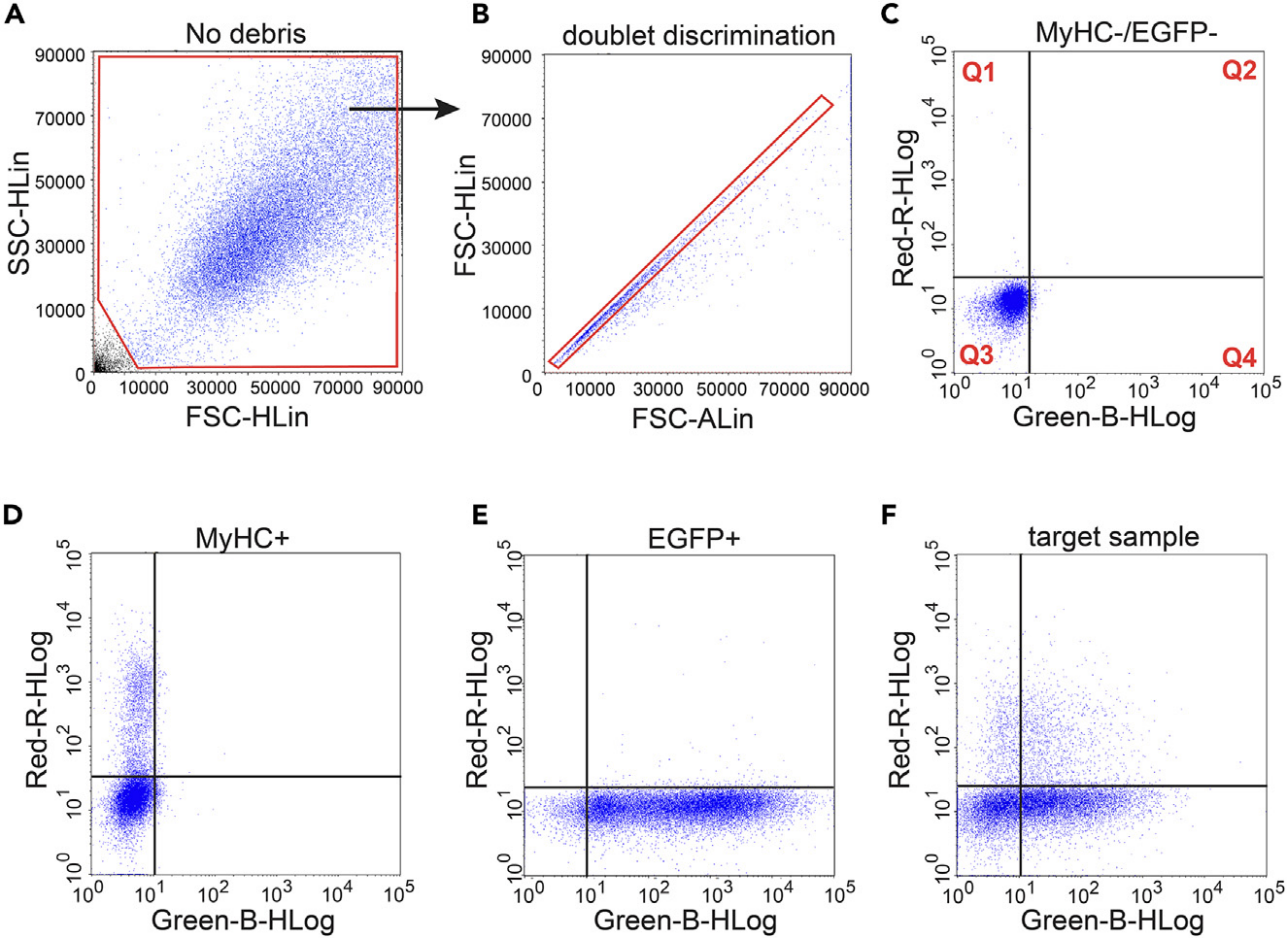
Flow cytometry gating strategy used to identify the differentiated MyHC+/GFP+ subpopulation of mEGFP-construct transfected C2C12 cells (A and B) Flow cytometry data dot plots with basic gates for debris exclusion (A) and exclusion of cell doublets in red (B). (C–E) All cells pass these basic gates and are then visualized in quadrants (Q1–Q4) as indicated. Samples used to adjust channel settings are double-negative cells (C), MyHC+ control cells (D) or GFP+ control cells (E). (F) Finally, target samples contain a differentiated subpopulation with MyHC+/GFP+ cells in Q2.

46. Always start the acquisition from the double-negative control. Gate around 30,000 cells based on their FSC and SSC properties to exclude debris or dead cells (Figure 2A).

**CRITICAL:** Maintain all here established instrument settings for detection channels and gates for all subsequent target sample acquisitions.

47. From this mixed population comprising myoblasts and myotubes, the doublets were then excluded using forward scatter height (FSC-H) and area (FSC-A) plotted on a linear scale (Figure 2B).

***Note:*** This is step is optional.

48. By applying these basic gates, singlet cells were visualized on a dot plot using the Green-B channel versus the Red-R channel in four quadrants (Figure 2C).

***Note:*** In GuavaSoft, quadrants cannot be set up during acquisition but only during analysis as in step 54. The double-negative control cells in quadrant 3 (Q3) have background MyHC-expression and do not express a GFP-variant construct, thus also in the Green-B channel showing only background autofluorescence of cells.

49. From the menu bar open the gain control panel and adjust the laser power for the 488 nm and 642 nm lasers as well as the gain for Green-B and Red-R channels.

**CRITICAL:** The gains must be set to display the cluster of double-negative cells in Q3, approximately around the level of ∼10^1^ relative fluorescence units (RFU) in both channels. This will allow the user to perform the analysis by considering all the events in Q3 as double-negative cells (Figure 2C).

50. Proceed by verifying that both the MyHC+ control and GFP+ control, are observed as expected i.e., MyHC+ cells along the vertical axis in the Red-R channel in Q1 (Figure 2D) and GFP+ cells along the horizontal axis in the Green-B channel in Q4 (Figure 2E).
51. Once all the controls are set, proceed with the acquisition of target samples, containing a subpopulation that is positive for GFP and MyHC in Q2 (Figure 2F).
52. For manual analysis of differentiation continue to part 7.
53. Alternatively, we recommend moving to part 8 for automated analysis of GFP-variant construct expressing cells using the FlowFate analysis software programmed in R.

### Part 7. Manual differentiation analysis on GuavaSoft 4.0 software

**Timing: ∼1 h**

In this **part,** we describe how to perform the manual analysis of the acquired flow cytometry data using the GuavaSoft 4.0 Software to quantify the percentage of differentiated cells in target samples. For GFP-variant expressing cells, we established an alternative automated analysis for this quantification as described in the subsequent part 8.

54. After implementing the basic gate settings from part 6 (Figures 2A and 2B), draw four analysis quadrants, by opening the ‘QuadStat Marker’ window.
55. Add a ‘New Stat’ using the ‘Count’ metric as in Figure 2C. Thus, cells in four different quadrants can be quantified (Figure 2C).

***Note:*** Procedures to visualize cells in dot plots and to quantify them within four quadrants or regions of interest can be found in all commercial flow cytometry analysis software.

56. Aided by the double-negative sample, position the QuadStat Marker at ∼10^1^ on both axes as the cluster of cells below ∼10^1^ in Q3 corresponds to double-negative cells (Figure 3).

**Figure 3:**
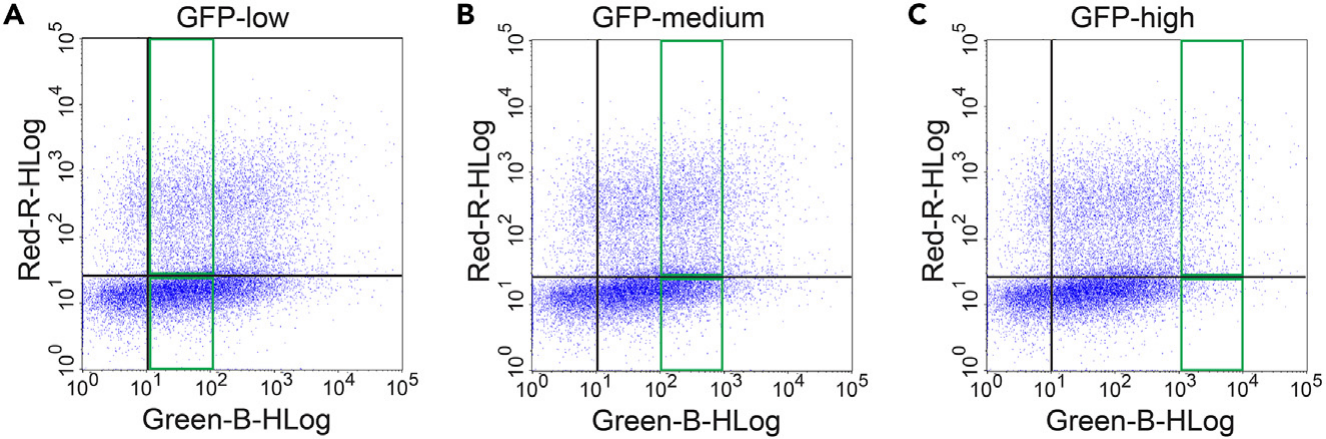
Gene dose dependent analysis of GFP-variant tagged constructs in three different GFP-expression bins (A–C) Plots of MyHC-labeled and GFP-variant tagged construct expressing cells from target samples. The green rectangles indicate the arbitrary GFP-low (A), GFP-medium (B) and GFP-high (C) expression bins of constructs.
57. Here, we also evaluate the effect a mEGFP-tagged gene of interest on differentiation. Cells exhibiting lowest GFP fluorescence between 10^1^ and 10^2^ RFU in the Green-B channel are assigned to the ‘GFP-low’ bin or window (Figure 3A).
58. Cells from this bin are considered MyHC+ if the signal in the Red-R channel was above ∼10^1^ threshold that was set up using the QuadStat marker.
59. Optionally, if a gene-dose dependent analysis is desired for the GFP-construct, draw additional bins.
60. Define between 10^2^ and 10^3^ RFU of the Green-B channel as ‘GFP-medium’ bin (Figure 3B) and between 10^3^ and 10^4^ RFU as ‘GFP-high’ bin (Figure 3C).
61. Set up the required GFP bins and identify MyHC+ and MyHC- cells as before.
62. In the target sample examples in Figure 4, cells were transfected with two K-Ras-constructs, N-terminally tagged with mEGFP, namely wild-type (wt) K-Ras (Figures 4A and 4B) or K-RasG12C (Figures 4C and 4D).

**Figure 4:**
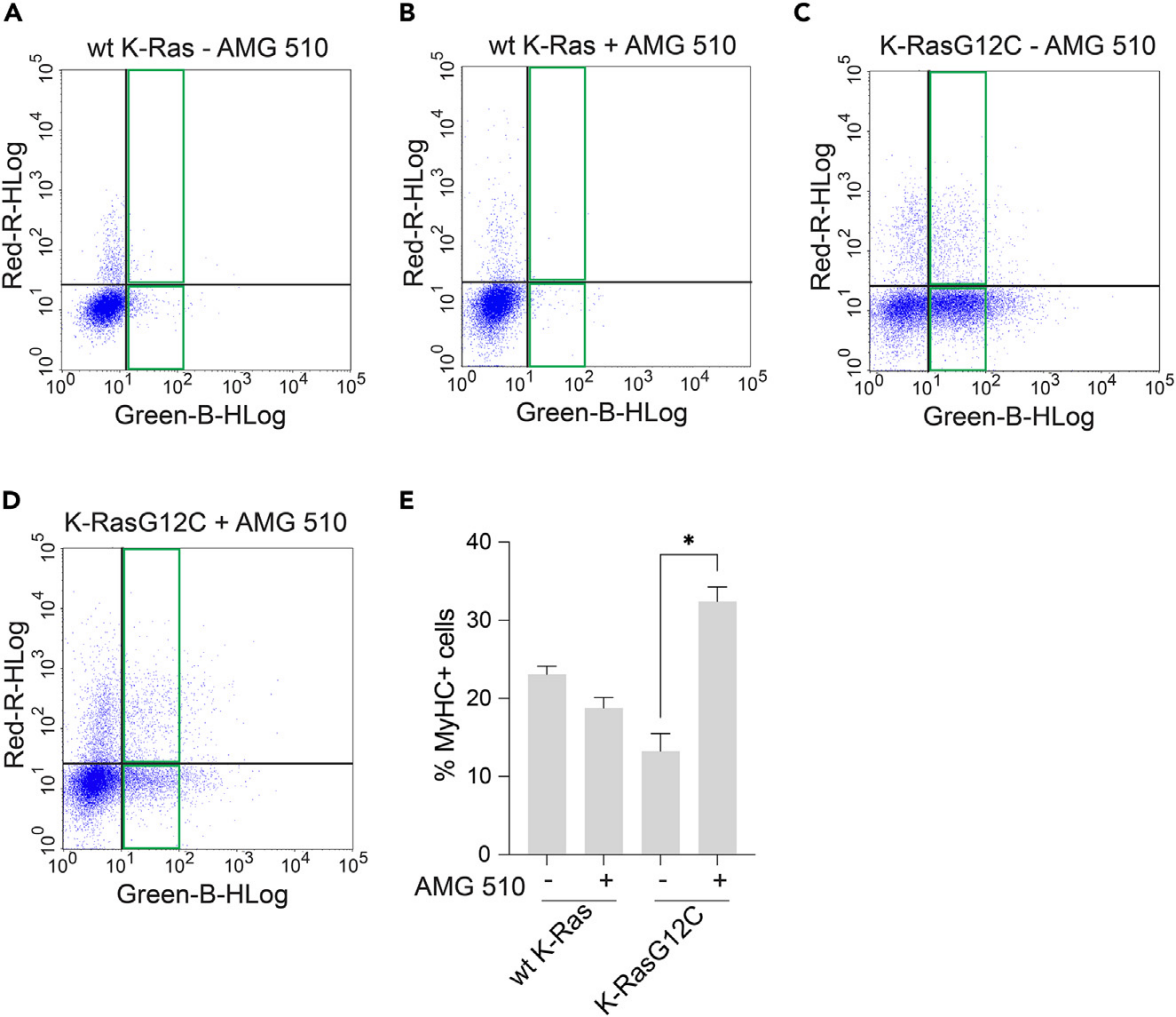
Example experiment showing differentiation outcomes of cells transfected with GFP-tagged K-Ras constructs and treated with a drug (A–D) Plots of MyHC-labeled and mEGFP-K-Ras constructs expressing cells from target samples: wild-type (wt) K-Ras with control 0.2% DMSO treatment (A); wt K-Ras treated with 3 μM AMG 510 (B); K-RasG12C with control 0.2% DMSO treatment (C); K-RasG12C treated with 3 μM AMG 510 (D). (E) Differentiation analysis results as percentage of MyHC+ differentiated cells in the GFP-low bin. Bar graph shows means ± SD. Conditions are as indicated; n = 3 independent biological repeats. Statistical comparison was done using Kruskal-Wallis test. ∗*P* < 0.05.
63. Samples were treated with either 0.2% DMSO in vehicle (Figures 4A and 4C) or K-RasG12C inhibitor AMG 510 (Figures 4B and 4D), and gated in the GFP-low bin.
64. Export the values of the count of MyHC+ and MyHC- cells from that GFP-bin to a graphing and analysis software, such as Microsoft Excel.
65. Calculate percentages of MyHC+ cells relative to the total number of GFP-low cells and plot them e.g., in a bar graph (Figure 4E).

### Part 8. Automated differentiation analysis with the custom-written FlowFate software in RStudio

**Timing: ∼15 min for first time users; ∼5 min thereafter**

We developed here FlowFate as a free, interactive R-based analysis software program that automates the quantitation of GFP-variant expressing C2C12 cell differentiation data as obtained by flow cytometry. Thus, it may be suitable for medium-throughput screening.

FlowFate can read flow cytometry data stored in flow cytometry standard (FCS) 3.0 format that originate from either single tube or 96-well plate acquisitions. Samples collected from a standard 96-well plate will be displayed by default with the corresponding well number. Prior to analysis, users should take note of the well numbers for each sample e.g., well A01 for double-negative sample etc.

FlowFate was developed by combining existing R packages (flowCore and flowWorkspace for FCS file import, data manipulation and gating, and ggcyto for visualization) into a customized workflow tailored to the analysis of differentiation data. FlowFate can quantify differentiated cells in up to three GFP-expression bins, thus enabling analysis of the gene dose-dependent effect of GFP-variant tagged/ coexpressing genes of interest. In the following, we employ as such a GFP-variant mEGFP.

First, the user has to perform the instrument set up essentially as described in part 6.

In FlowFate we have then eliminated manual gating steps by using pre-defined settings to exclude debris (Figure S4B) and identify GFP+ cells during the ‘Curate’ step. GFP-thresholding is achieved by utilizing the ‘quantile gate’ from openCyto. Quantile gates are used to determine a threshold below which 99% of cells are found. This GFP-threshold value is obtained by averaging the GFP-channel data from the double-negative and MyHC+ control samples analogous to what was described in part 6, as depicted in Figure S4C. The GFP-threshold is then applied to all samples during the ‘curation’ step to analyze only GFP+ events. It also sets the lower limit of the GFP-low bin. To examine the gene dose-dependent effect of the GFP-variant tagged/ coexpressing gene of interest, up to three GFP-expression bins can be defined (GFP-low, -medium and -high).

The ‘Split peaks’ command automatically plots histograms of the MyHC-channel within each user-specified GFP-bin for every sample. We use a ‘peak-splitting’ algorithm from openCyto, which determines the intersection point between two population peaks and automatically creates a gate including intensities above this cutpoint (the cutpoint is depicted as a red line in Figure S5D). The two distinct subpopulations are MyHC- (intensity peak on the left of the red line) and MyHC+ (intensity peak on the right of the red line) (Figure S5D). The latter population represents differentiated C2C12 myocytes.

66. One time installation of RStudio and FlowFate.
  a. To run the app in R, install R and RStudio from: https://posit.co/download/rstudio-desktop/. R should always be installed before RStudio, as indicated on the website.
    ***Note:*** Screenshots from the installation steps of R (Figures S1A–S1D) and RStudio (Figure S1E) are provided for illustration.
  b. Launch RStudio, and click ‘File’ > ‘New File’ > ‘R script’ (Figures S1F and S1G).
  c. In the top left window of this new R script, type the following commands and execute by pressing ‘command + return’ for Mac users, or ‘ctrl + enter’ for Windows user after each command.
  d. Install the ‘remotes’ and ‘BiocManager’ packages with this command:
    >install.packages(c("remotes", "BiocManager"))
    ***Note:*** Red text appears in the console; wait until text spooling stops, which takes several seconds (Figure S2A).
  e. Install Bioconductor dependencies with this command:
    >BiocManager::install(c("ggcyto","flowWorkspace","flowCore "))
    ***Note:*** The following question pops up in the console: ‘Update all/some/none? [a/s/n]’. In the console, answer ‘a’ and execute by pressing ‘command + return’ (Figure S2B).
  f. Install Bioconductor dependencies with this command:
    >remotes::install_github("RGLab/openCyto")
    ***Note:*** Red text appears in the console; wait until text spooling stops, which takes several seconds. Another question pops up ‘These packages have more recent versions available. It is recommended to update all of them. Which would you like to update?
    All.
    CRAN packages only.
    None.
    deldir (1.0–6 -> 1.0–9 ) [CRAN].
    ncdfFlow (2.45.0 -> 2.46.0) [CRAN].
    rrcov (1.7–2 -> 1.7–3 ) [CRAN].
    flowWorks... (4.11.1 -> 4.12.0) [CRAN]’.
    In the console, answer ‘1’ and execute by pressing ‘command + return’ (Figure S2C).
  g. Install FlowFate package with this command:
    >remotes::install_github("maximesunnen/flowFate@∗release")
    ***Note:*** Red text appears in the console; wait until text spooling stops, which takes several seconds. Another question pops up ‘These packages have more recent versions available. It is recommended to update all of them. Which would you like to update?
    All.
    CRAN packages only.
    None.
    jsonlite (1.8.4 -> 1.8.5) [CRAN].
    golem (0.4.0 -> 0.4.1) [CRAN].

In the console, answer ‘1’ and execute by pressing ‘command + return’. Installation is now complete as indicated by text boxed in the blue Figure S2D.

***Note:*** Installation of packages needs to be done only once, before running FlowFate for the first time. To verify whether a new version of FlowFate is available, the user should check the version number on the GitHub page of FlowFate (www.github.com/maximesunnen/flowFate). To download the new release, the command in step 66.g (Install FlowFate package) must be re-executed.

67. Running the differentiation analysis using FlowFate.
  a. Within R-studio, launch the FlowFate software app with this command (Figure S2E):
    >flowFate::run_app()
    A new window will open within RStudio which will launch FlowFate.
  b. Click ‘Open in Browser’ to launch the software on the web browser (Figure S3A). While the display is enabled by the web browser, all processing happens on your local computer.
  c. Select the data format in which the .fcs files are stored.
    i. To upload a merged file containing all controls and target sample data, select ‘Single, merged FCS file’. Click ‘Browse’ to select the file (Figure S3A).
    ii. For uploading individual .fcs files containing control and target sample data separately within one folder, select ‘Folder with individual FCS files’. Click ‘Browse’ and select the folder containing all the files that were acquired within the same experiment (Figure S3B).
      ***Note:*** For first time users, it is recommended to study the analysis pipeline, using a demo dataset by clicking ‘Import demo data’ (Figure S3C). All the samples in the demo dataset are described in Table S1.
  d. Using your own .fcs files, click ‘Submit’ to confirm your selection and to view your uploaded data (Figure S3D).
  e. Continue with either your own or demo data.
    i. At the top in the menu bar, click ‘Explore’ (Figure S3E).
    ii. On the left, select the plot type, the scale (linear or biexponential) and channels to display on the X- and Y-axes.
      ***Note:*** For example, select ‘linear’ scale to plot FSC on the X-axis and SSC on the Y-axis (Figure S3F).
  f. Since the fluorescent markers are visualized on a logarithmic scale, select ‘biexponential’ for instance for the GFP-channel (here ‘GRN.B.HLin’) to plot the GFP+ cells on the X-axis and the MyHC-channel (here ‘RED.R.HLin’) to plot MyHC+ cells on the Y-axis (Figure S3G).
    ***Note:*** The plotted data is raw and uncurated, hence it also contains debris.
  g. On the menu bar, click ‘Curate’ (Figure S4A).
    i. Select the channel names from your instrument used for GFP- and MyHC-detection.
    ii. Select here in the demo dataset for ‘GFP channel’ ‘GRN.B.HLin’ and for the ‘MyHC channel’ ‘RED.R.HLin’.
  h. Select your control samples corresponding to ‘double-negative control’ and ‘MyHC+ control’ as described in part 6.
  i. ‘Start curation’ automatically sets up a pre-defined gate to remove cellular debris, based on the double-negative- and MyHC+ -control samples.
  j. Make sure you have a nice single-cell suspension (Figure 2B), as doublet-discrimination is not implemented here.
  k. Navigate through the ‘NonDebris gate’ tab to display results from this part of the curation process (Figure S4B).
  l. Curation in addition sets a GFP-threshold. Cells above this threshold are GFP+ and cells below this threshold are GFP-.
    ***Note:*** This function permits the exclusion of untransfected or autofluorescent cells from the analysis.
  m. Click the ‘GFP gate’ tab to display results from that part of the curation process (Figure S4C).
  n. Next, GFP-expression level bins are set up.
    i. On the menu bar, click ‘Gate’ and then select ‘Add GFP bin’ to add at least a GFP-low bin (Figure S5A).
    ii. Then specifying the desired intensity range of that bin (Figure S5B), click ‘Confirm’ (Figure S5A).
      ***Note:*** The first value displayed in the GFP-low bin corresponds to the automatically applied GFP-threshold value and it should remain unaltered.
  o. Optionally, if a gene-dose dependency is intended to be analyzed, you can set two additional bins, GFP-medium and -high.
    i. Click on ‘Add GFP bin’ and specify the desired intensity range (Figure S5A).
  p. For the demo data, specify three GFP bins: GFP-low = GFP channel intensities from 24.1 to 100 RFU; GFP-medium = GFP channel intensities from 101 to 1000 RFU; GFP-high = GFP channel intensities from 1001 to 10000 RFU.
  q. Click ‘Confirm’ to set each range (Figure S5A). You can reset the GFP-bin assignment by clicking the ‘Reset bins’ button (Figure S5B).
  r. After FlowFate has switched to the ‘Split peaks’ tab, click ‘Split’ (Figure S5C).
    **CRITICAL:** This important step automatically identifies the MyHC- and MyHC+ subpopulations within the defined GFP-bins.
  s. After clicking ‘Split’, the app automatically opens the ‘Plot’ panel (Figure S5D). You can browse data in the window.
    ***Note:*** Target samples ‘03_G12C_DMSO’ to ‘04_G12C_AMG510’ within the demo dataset were considered for the subsequent steps. ‘03_G12C_DMSO’ comprises cells transfected with K-RasG12C treated with 0.2% DMSO and ‘04_G12C_AMG510’ represents cells transfected with K-RasG12C treated with 3 μM AMG 510 (Table S1).
  t. In the ‘Split peaks’ panel, individual datasets can be selected to display MyHC intensity distribution.
    ***Note:*** Multiple selections are possible by clicking on them to facilitate sample comparisons (Figure S5E).
  u. To store your analysis for processing, click ‘Export’ on the menu bar (Figure S5F).
  v. Click ‘Show population statistics’ to get a table with population data necessary for statistical analysis outside of FlowFate.
    ***Note:*** This table contains three columns, (1) sample name (sample), (2) population name (pop), (3) population count (count). ‘Count’ corresponds to the number of events in the respective population.
    The nomenclature used to unambiguously identify the different populations is explained in Table 1.

**Table 1:** Hierarchical dataset description

Population name (pop)	Definition
root	Total events (raw import data)
/NonDebris	Intact cells, thresholded for debris exclusion
/NonDebris/GFP+	Intact cells with GFP intensities above the GFP-threshold; removed untransfected or autofluorescent cells *GFP+ cells in Q2 and Q4 (*Figure 2F*)*
/NonDebris/GFP+/GFP-low *(-medium; -high)*	Intact cells with GFP intensities inside the GFP-low (-medium; -high) bins *GFP-low bin (*Figure 3A*)*
/NonDebris/GFP+/GFP-low (-medium; -high)/MyHC+	Intact cells with GFP intensities inside the GFP-low (-medium; high) bins and differentiated with high MyHC expression *MyHC+/GFP+ cells in GFP-low bin of Q2* (Figure 3A)
  w. Specify your filename and click ‘Download’ to export the data as a .csv file (Figure S5F).
  x. To calculate the fraction of differentiated cells in the GFP-low bin, divide data from /NonDebris/GFP+/GFP-low/MyHC+ count (MyHC+ cells in GFP-low bin) by /NonDebris/GFP+/GFP-low count (total number of GFP+ cells in the GFP-low bin).
  y. Apply this processing rationale to all of your target samples.

## Expected outcomes

We have established an analysis pipeline to accurately quantify C2C12 cell differentiation using flow cytometry (Figure 1). We describe the cultivation and differentiation of C2C12 cells and their subsequent fluorescent labeling for flow cytometry-based analysis of the expression of the differentiation marker MyHC (Figure 2). Our detachment and gating procedure ensures that also myotubes are analyzed, which we have validated by applying a differential trypsinization protocol^11^ (Figures S6A and S6B). Genetic perturbations of C2C12 cells can be quantitated after transient expression of GFP-tagged genes of interest. Note, however, that the fraction of GFP+ cells can vary significantly depending on the conditions (Figure S6C). In addition, it may be possible to analyze genes of interest coexpressing a GFP-variant from an IRES-element or via a P2A-autocleavage site (Figures S7A and S7B).^12^ Based on our data, we however recommend direct tagging if possible.

By gating for different GFP-expression levels, a gene-dose-dependence analysis can be performed (Figure 3). Comparison of the differentiation outcomes of non-transfected cells with cells expressing the wild-type of a gene at low, medium or high levels, will allow assigning non-perturbing, near native expression level conditions. We furthermore illustrate the potential of our pipeline for drug discovery in cancer, by showing how drug treatment restores normal differentiation that was inhibited by the transient expression of an mEGFP-tagged oncogenic K-Ras construct (Figure 4).

**Figure 5:**
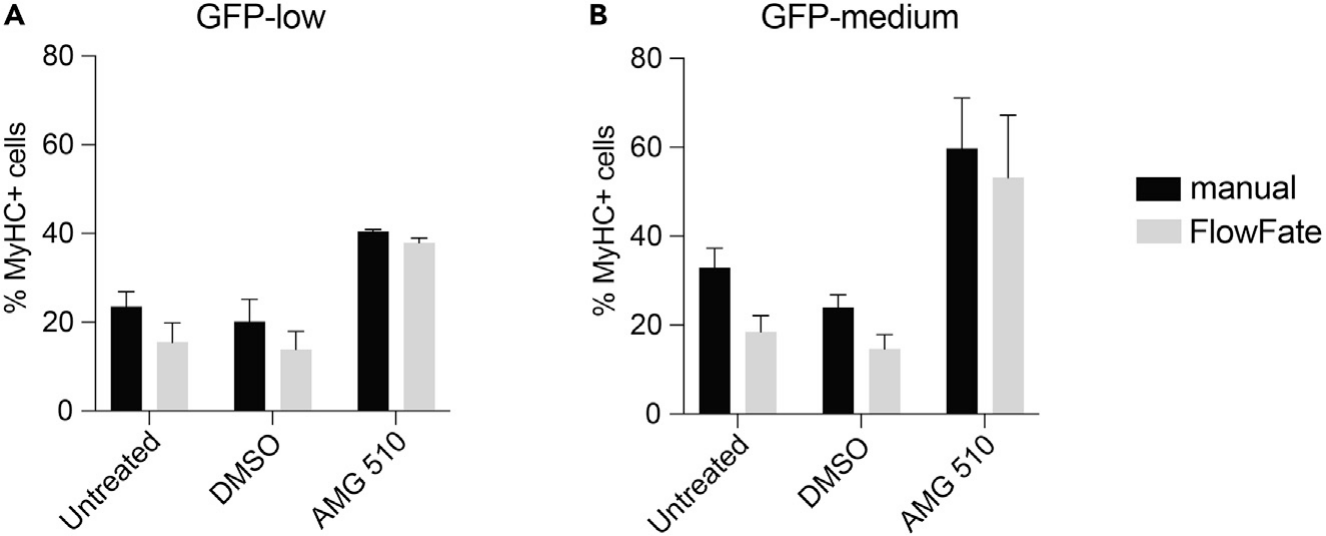
Comparison of MyHC+ quantifications obtained by manual analysis with GuavaSoft 4.0 Software (part 7) or automated analysis using FlowFate (part 8) (A and B) C2C12 cells were transiently transfected with mEGFP-K-RasG12C and treated as indicated. For sample descriptions see Table S1. Bar graphs show the percentages of MyHC+ cells quantified manually or using FlowFate in ‘GFP-low’ (A) and ‘GFP-medium’ (B) bins. Data are mean ± SD of n = 2 independent biological repeats. Comparisons between manual and FlowFate quantifications were non-significant. Statistical comparison was done using Mann-Whitney test.

Importantly, we implement FlowFate, an R-based automated analysis solution for flow cytometry derived differentiation data of C2C12 cells that express a GFP-variant construct. This software greatly increases the speed of data analysis, thus allowing the comparison of multiple conditions, such as diverse genetic constructs and drugs. Our comparison with a manual analysis shows that the automated analysis with FlowFate performs indistinguishably (Figure 5). By automating the analysis, FlowFate does not only save manual processing time, but also eliminates manual bias or errors.

We foresee a significant potential of our analysis pipeline to assess the effect of drugs on oncogene transformed C2C12 cell differentiation. As such it will be an important complementation to standard cancer cell proliferation and viability assays.

## Limitations

This protocol has been optimized for flow cytometry data analysis of differentiated C2C12 cells that are identified by their MyHC+ labeling. The analysis of differentiation with other markers is potentially possible with this method but would require a comprehensive analysis of their suitability for flow cytometry-based analysis.

Transfection efficiencies of C2C12 cells are typically several-fold lower than for HEK 293T cells, which may complicate or obviate the analysis of certain genetic constructs that express poorly. Similarly, the GFP-signal based analysis of different gene doses of GFP-variant construct transfected cells may be different or not achievable on other flow cytometers, as the dynamic range for the detection of the GFP-fluorescence may be insufficient.

In FlowFate, debris are excluded with a fixed gate (Figure S4B), where gate boundaries are predefined, and users are not able to manually change them. The setup of gates as described in part 6 (Figure 2) therefore must be strictly followed. However, advanced R users can manually adjust these gate boundaries by changing the respective gating matrix in the source code.

## Troubleshooting

### Problem 1

Cells poorly differentiate i.e., MyHC+ fractions are below 5% for control samples that were incubated in low serum medium for 3 days (part 7 or 8).

### Potential solution

• Conditions for serum switching to low serum medium are optimal when cells reach a confluency of 90%. This should be confirmed by microscopic observation before switching the serum.
• Batches of cells with a higher passage number differentiate poorly as compared to lower passages. Using a batch with a passage number below ten is highly recommended.
• Treatment with some drugs or the transfection of certain constructs may result in cell toxicity and a reduced differentiation output. Observing cell morphology via microscopy or testing for viability/ apoptosis may help to establish conditions without cytotoxicity.

### Problem 2

Flakes and aggregates appear in the cell suspension after trypsinization (step 28).

### Potential solution

• Cells cultured for three or more days in low serum medium tend to aggregate upon longer incubation times with trypsin. In such cases, it is advisable to leave cells with trypsin for only 2 min at 22°C–25°C and immediately start pipetting and detaching the cells.
• Vigorous pipetting at this stage and avoiding longer incubations with trypsin would avoid this issue.

### Problem 3

Cells tend to die after transfection (part 2).

### Potential solution

• Cell confluency should be ideally 50%–60% on the day of transfection. If cells are at a lower confluency, it is advisable to wait for cells to attain the appropriate confluency before transfecting.
• The incubation time with jetPRIME could be reduced from 4 h to 3 h.
• DNA concentrations greater than 1 μg/ mL medium may also result in toxicity.

### Problem 4

FlowFate crashes (part 8).

### Potential solution

• Do not start curation before the upload of the .fcs file is complete.
• Restart the app and repeat the analysis by uploading again the .fcs file.

### Problem 5

MacOS users are unable to install openCyto or other packages (part 8).

### Potential solution

• A code compiler is probably missing from the user’s computer and needs to be installed before running the code.
• A common compiler for MacOS is Xcode (developer tools), which can be installed from the app store.
• Then RStudio can be restarted to run the commands in part 8.

## Resource availability

### Lead contact

Further information and requests for resources and reagents should be directed to and will be fulfilled by the lead contact, Daniel Kwaku Abankwa (daniel.abankwa@uni.lu).

### Materials availability

This study did not generate new unique reagents.

## Data Availability

The source code for the app and the sample data files are freely available on GitHub at www.github.com/maximesunnen/flowFate.
