# Supplementary material for: A flow-cytometry-based pipeline for the rapid quantification of C2C12 cell differentiation
Source: STAR Protoc. 2023 Oct 10;4(4):102637. doi: 10.1016/j.xpro.2023.102637 (PMC10568640; doi:10.1016/j.xpro.2023.102637)
Supplement: Document S1. Figures S1–S7 and Table S1 [file mmc1.pdf]

Supporting Information

Supplementary Tables

Table S1: Control and target samples included in the demo dataset, related to Part 8.67.c. This table describes the properties of samples provided as demo data.

Name	Type	Definition
00_Double-negative	negative control	Untransfected cells cultured in high serum, immunolabelled with MyHC antibody.
01_MyHC_plus_only	positive control	Untransfected cells cultured under low serum, to induce differentiation and MyHC expression, immunolabelled with MyHC antibody.
02_G12C_Untreated	target	mEGFP-K-RasG12C transfected cells cultured in low serum, immunolabelled with MyHC antibody.
03_G12C_DMSO	target	mEGFP-K-RasG12C transfected cells cultured in low serum, treated with 0.2 % DMSO vehicle control, immunolabelled with MyHC antibody. NB: Oncogene expression blocks differentiation.
04_G12C_AMG510	target	mEGFP-K-RasG12C transfected cells cultured in low serum, treated with 3 μM AMG 510, immunolabelled with MyHC antibody. NB: The inhibitor restores the differentiation.

Supplementary Figures

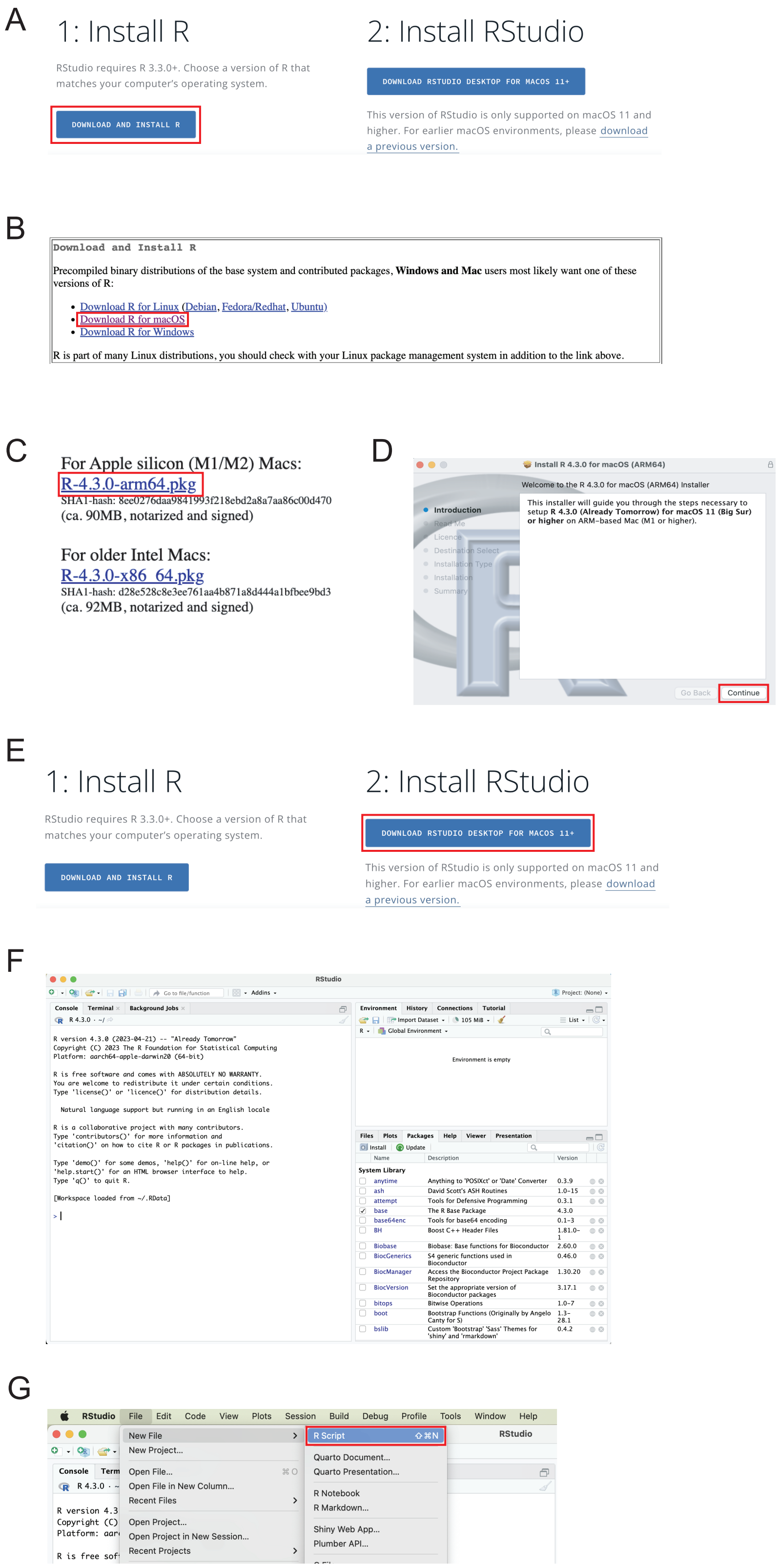

Figure S1: Screenshots of R and RStudio installation process, related to Part 8.66.a,b.

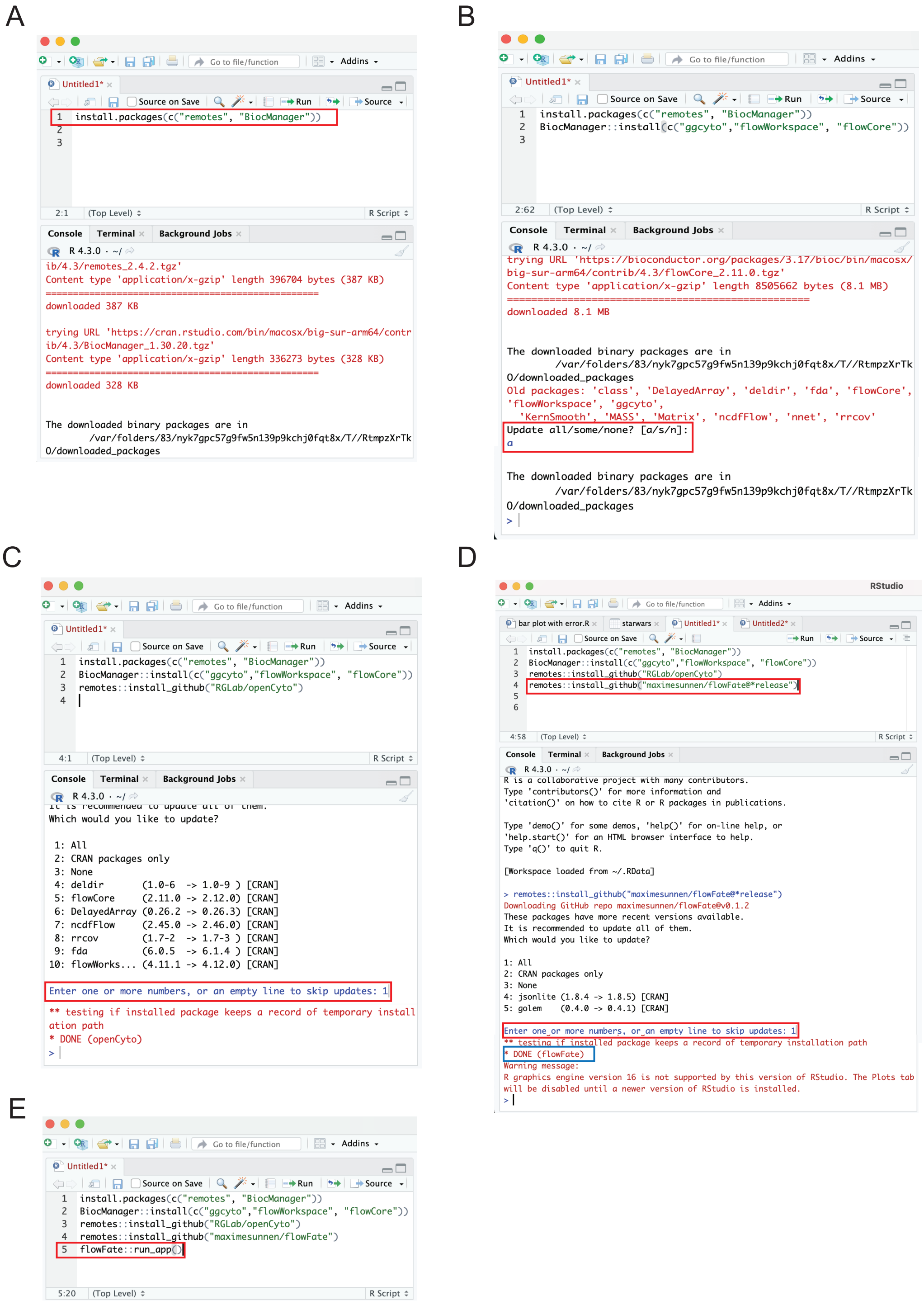

Figure S2: Screenshots of the installation of four additional R packages (ggcyto, flowWorkspace, flowCore and openCyto), related to Part 8.66.d-g and Part 8.67.a.

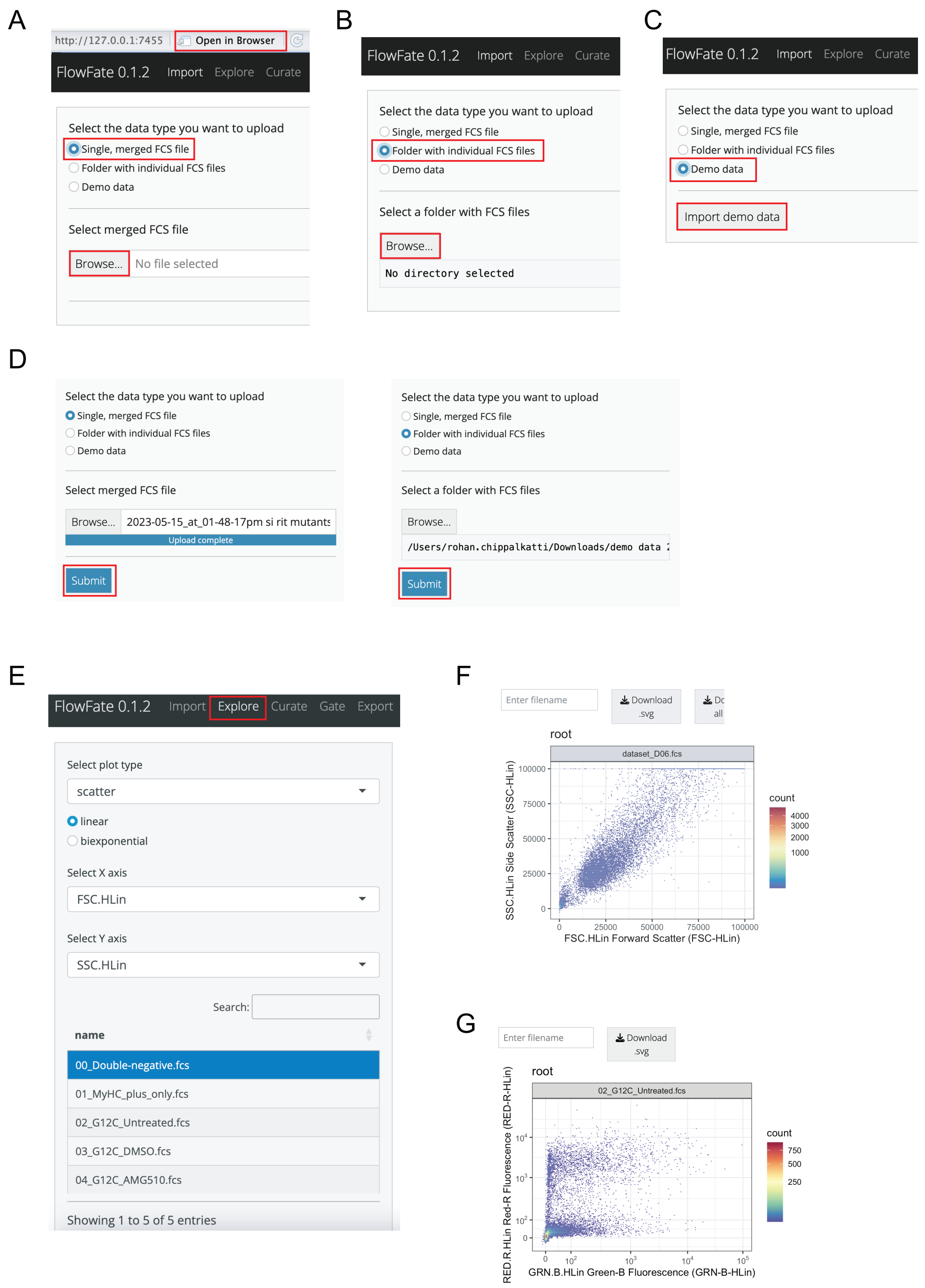

Figure S3: Screenshots of FlowFate FCS file import, related to Part 8.67.b-f. Import of merged or individual FCS files and their visualization is depicted.

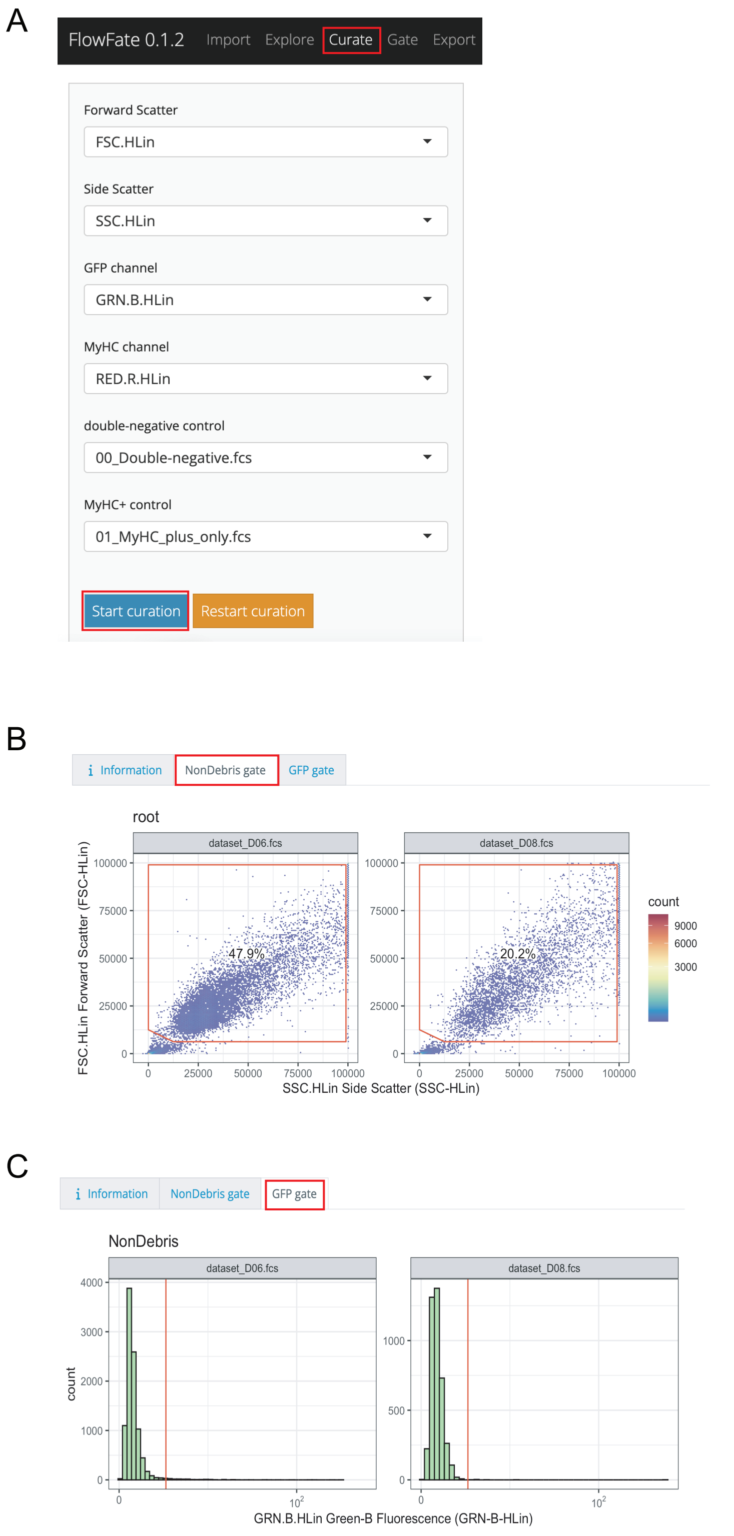

Figure S4: Screenshots of curation steps in FlowFate, related to Part 8.67.g-m. For the selected samples, FlowFate automatically applies thresholds to exclude debris and GFP- events.

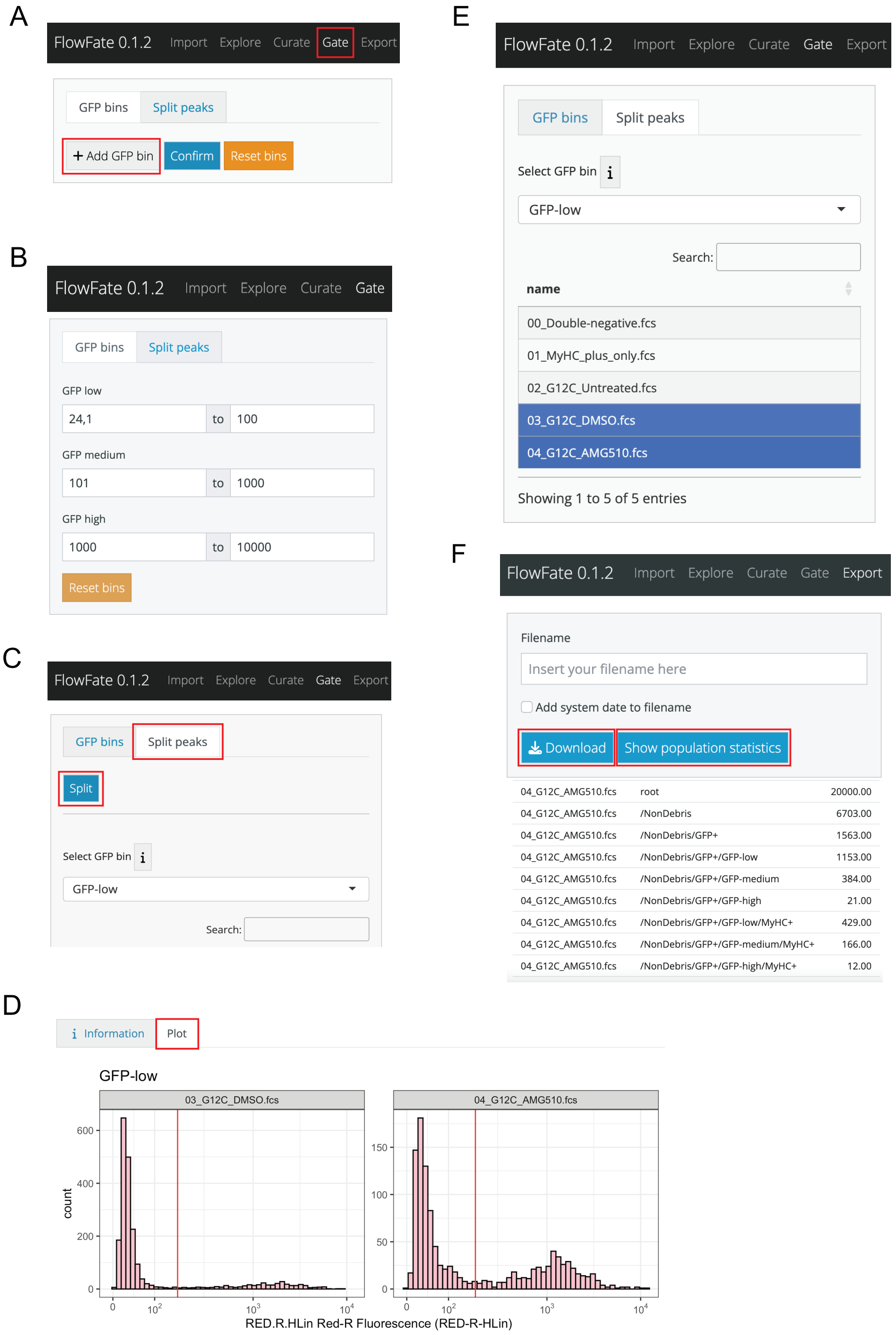

Figure S5: Screenshots of binning steps for GFP+ cells, related to Part 8.67.n-w.

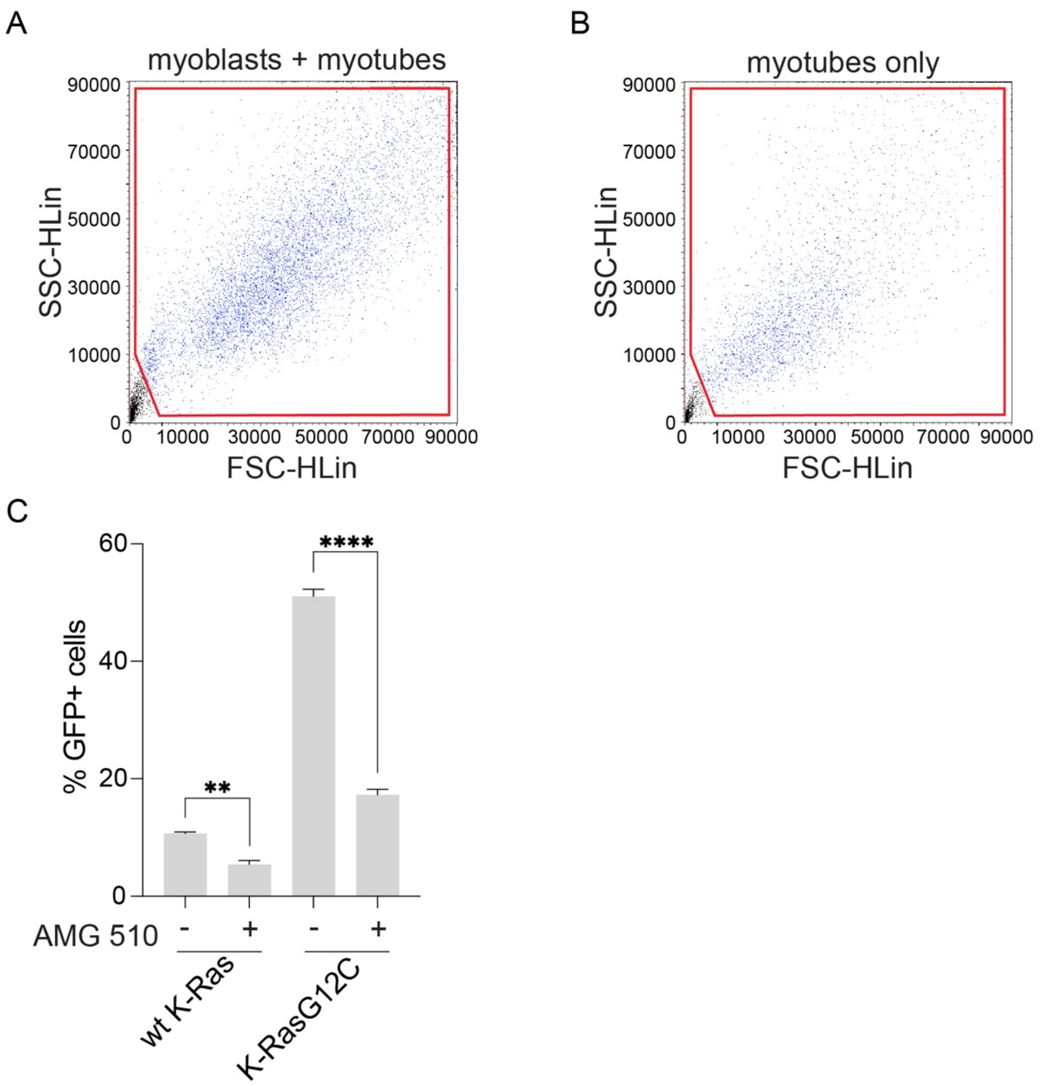

Figure S6: Validation for inclusion of myotubes in the first gate and fraction of GFP-positive cells by sample, related to the Expected Outcomes section. (**A,B**) Representative dot plots illustrating FSC- and SSC-characteristics of cells cultured in low serum for three days. After 3 days in low serum, the C2C12 cell population is mixed, containing myoblasts and myotubes (A). It is possible to enrich for myotubes by differential trypsinization. Briefly, cells were incubated with 0.025 % trypsin EDTA for only 1 min with intermittent tapping of the culture plate. Myotubes detach more easily and can thus be collected first (B). Cells were then prepared as described up to **part 5.35**. As can be seen, both the original culture and the myotube enriched fraction essentially overlap and thus fall within the initial gate for intact cells within FlowFate (red). (**C**) Data from **Figure 4E** were analyzed for the fraction of GFP+ cells relative to the total number of intact cells. Means ± SD are plotted. Statistical analysis was done using one-way ANOVA. ***P*<0.01; *****P*<0.0001.

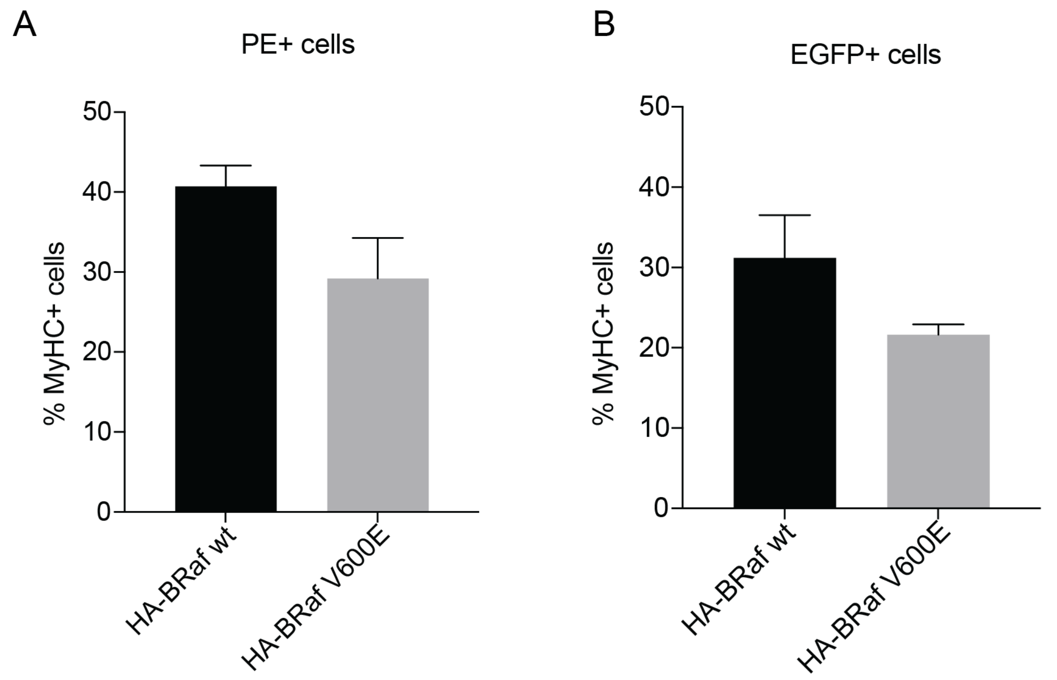

Figure S7: Differentiation analysis of HA-tagged BRaf variants that are coexpressed with EGFP from an IRES-element, related to the Expected Outcomes section. (**A,B**) HA-BRaf wild-type (wt) or oncogenic HA-BRafV600E expressing cells were labelled with a PE-conjugated anti-HA-tag antibody. EGFP was coexpressed from an IRES-element. Differentiated MyHC+ cells were quantified from either PE+ cells (A) or EGFP+ cells (B), following the specified transfection protocol (**part 2**). As expected, we do see that in both cases the oncogenic HA-BRafV600E expression blocks differentiation as compared to the wild-type counterpart. However, given the quantitative differences, we recommend using directly tagged constructs or further optimization of this mode of analysis. Means ± SD are plotted; n = 3 independent biological repeats.
